# Would the field of cognitive neuroscience be advanced by sharing functional MRI data?

**DOI:** 10.1186/1741-7015-9-34

**Published:** 2011-04-08

**Authors:** Kristina M Visscher, Daniel H Weissman

**Affiliations:** 1 Department of Neurobiology, University of Alabama, Birmingham, AL 35294, USA; 2 Department of Psychology, University of Michigan, Ann Arbor, MI, 48109, USA

## Abstract

During the past two decades, the advent of functional magnetic resonance imaging (fMRI) has fundamentally changed our understanding of brain-behavior relationships. However, the data from any one study add only incrementally to the big picture. This fact raises important questions about the dominant practice of performing studies in isolation. To what extent are the findings from any single study reproducible? Are researchers who lack the resources to conduct a fMRI study being needlessly excluded? Is pre-existing fMRI data being used effectively to train new students in the field? Here, we will argue that greater sharing and synthesis of raw fMRI data among researchers would make the answers to all of these questions more favorable to scientific discovery than they are today and that such sharing is an important next step for advancing the field of cognitive neuroscience.

## What is functional MRI?

Functional MRI is a non-invasive technique for determining the neural correlates of mental processes in humans and other animals. During the past two decades, this technique has become a leading method in the field of cognitive neuroscience that has brought about revolutionary changes in our understanding of brain-behavior relationships [reviewed, e.g., in [1]]. Nevertheless, functional MRI studies yield tremendous quantities of multidimensional data and are expensive to conduct. This raises the question of whether greater sharing of fMRI data would allow the field to get more 'bang for its buck' from each data set.

## Potential benefits of sharing fMRI data

What if the raw anatomical and functional images from every published fMRI study were freely available online? In our view, such a scenario would benefit the field of cognitive neuroscience in numerous ways [see also [2,3]]. First, it would allow researchers to quickly establish the reproducibility of a new finding and to test hypotheses using much larger data sets. Second, it would allow researchers with complementary expertise to provide multiple characterizations of the same data. Third, it would enhance the training of new cognitive neuroscientists.

## Quickly establishing the reproducibility of a finding

Replication is important in every scientific discipline for showing that an effect is real (that is, it is not an artifact of a particular procedure or analysis). If data sharing were more prevalent, then it would often be possible to quickly determine whether a new finding is reproducible. For example, one could re-analyze similar data from multiple laboratories to determine whether, and under what conditions, the effect is present. This would speed the community's ability to assess the significance of a new finding and, if warranted, plan future studies. For instance, it might speed the community's ability to assess whether a new finding is relevant to treating a neurological disorder - a top priority of various funding agencies (for example, the United States National Institutes of Health). In the absence of extensive data sharing, researchers sometimes reanalyze multiple data sets from their own laboratories [e.g., [4]]: a useful but more limited approach.

## Allowing multiple researchers with complementary expertise to characterize the same data

The field of cognitive neuroscience is becoming increasingly specialized. Both the nature of processes being investigated (for example, cognitive, affective, social, cultural, and so on) and the types of fMRI methods used to identify the neural mechanisms underlying these processes (for example, blocked designs, event-related designs, mixed designs, functional connectivity, effective connectivity, pattern classification, and so on) have exploded in the past ten years. Given such specialization, any single researcher is unlikely to know all of the hypotheses (and methods) that could be tested (and employed) in a particular study. Greater sharing of fMRI data could thus allow more hypotheses to be tested with a given data set, a desirable outcome given the high cost of obtaining fMRI scans from each study participant (typically hundreds of dollars per hour).

Data sharing could also promote synergistic activities between researchers that would not otherwise occur. Two cognitive neuroscientists with complementary methodological expertise (for example, functional connectivity and pattern classification techniques) might work together to characterize the relationship between distinct neural measures of a particular cognitive process (for example, attention). Additionally, cognitive neuroscientists might benefit more extensively from analytic techniques developed in other fields (for example, engineering or mathematics) that enable entirely new classes of hypotheses to be tested. The application of pattern classification methods to fMRI data is a timely example of how a transplanted mathematical technique has greatly influenced cognitive neuroscience research [5]. If data sharing were more prevalent, then it would be easier for scientists in other fields to develop new methods for analyzing fMRI data. This could speed the rate at which those methods enable new discoveries in cognitive neuroscience (Text Box).

More extensive data sharing would also aid researchers who do not have the resources to conduct their own fMRI studies. A shortage of resources might arise from a lack of access or expertise with regard to special populations. For example, studies of clinical disorders are often harder to conduct than studies of healthy controls because they require expertise related to recruiting, diagnosing, and interacting with patients. A dearth of resources could also arise from a temporary lack of funding. Indeed, the percentage of grant applications funded by the National Institutes of Health (NIH) and the National Science Foundation (NSF) varies dramatically with the size of the federal budget. When funding percentages are low, greater data sharing would facilitate the ability of talented researchers to continue investigating intriguing brain-behavior relationships.

Influential government organizations also see the value of data sharing. The Organization for Economic Co-operation and Development, which includes officials from 30 democracies across the globe, states that 'access to research data increases the returns from public investment in this area; reinforces open scientific inquiry; encourages diversity of studies and opinion; promotes new areas of work and enables the exploration of topics not envisioned by the initial investigators' [6]. Similarly, the NIH, a major funding agency in the United States, views data sharing as 'essential for expedited translation of research results into knowledge, products, and procedures to improve human health' [7]. In short, there is a widespread perception that data sharing can advance science by allowing researchers with complementary expertise to characterize the same data. Nonetheless, data sharing is not yet standard practice in many fields [8], including cognitive neuroscience.

## Enhancing the training of new cognitive neuroscientists

Training new students in fMRI methodology is crucial to the future of cognitive neuroscience. Given that numerous methods exist for analyzing fMRI data (see above), such training would ideally involve reanalyzing publicly available data sets that map onto published studies in the literature. By comparing the results of their re-analyses to the published data, new students could check whether they are performing the analyses correctly. More fundamentally, they would learn how various methods give rise to different findings in the literature. Such training is especially important given that many students find themselves in laboratories that use a restricted range of fMRI methods.

These intuitions about the usefulness of publicly available data sets are borne out by experience. For example, DHW uses a publicly available data set to teach event-related fMRI regression analyses in a functional MRI methods course [9]. At present, however, publicly available data sets do not cover the full range of analyses that are reported in the literature. Thus, greater data sharing would facilitate educators' ability to teach an even wider variety of fMRI analyses.

## Potential pitfalls of sharing fMRI data

Given that data sharing has so many potential benefits, why is it not more prevalent? There are likely many reasons. First, researchers may dread the time needed to organize data from an fMRI study into a standardized format and transfer it to an online repository. Second, they may wonder whether subject confidentiality can be maintained. Third, they may be concerned that other researchers could reanalyze their data and publish important results before they can (for example, from a data set that is expected to yield multiple papers). Fourth, they may worry about the results of a reanalysis suggesting that a previously published finding is inaccurate, whether or not that is truly the case. In short, despite the potential benefits of data sharing, outlined above, there are many reasons why researchers may choose not to share their fMRI data [10]. We now discuss these potential barriers to sharing fMRI data and suggest some ways to overcome them.

## Organizing and depositing data in an online repository and maintaining subject confidentiality

Some of these issues may not be too difficult to address. First, researchers already have an incentive to archive their data sets in a standardized format, if only to ensure that they can readily access their own data at some later time. Thus, if a universal standard could be agreed upon, and was not overly time-consuming to implement, then researchers would be more likely to adopt it. Discussions concerning the nature of such a standard format for raw data are ongoing in conference proceedings [for example [11,12]], and in online forums such as the 'Neuroimaging Data Access Group' (http://www.nidag.org/) and the 'International Neuroinformatics Coordinating Facility' (http://www.incf.org/). Updating popular fMRI analysis packages with programs that archive data in a standard format could facilitate the community's transition by decreasing the time required to move data to a standardized format. Second, the difficulties associated with placing archived data into an online repository might be eased with user friendly, high speed interfaces that make data organization and/or transfer less tedious. Third, it is possible to de-identify functional and anatomical scans to maintain subject confidentiality [e.g., [13]]. For these reasons, such issues are unlikely to form insurmountable barriers to data sharing.

On the other hand, it may be more difficult to ensure that data within a repository is readily searchable. This requires a consistent vocabulary (that is, a set of keywords) for describing each study. For example, any study involving a manipulation of spatial attention should be tagged with a key word like 'SPATIAL ATTENTION.' However, researchers may not consistently use the same keywords, even when describing similar data sets [3,14]. There is an ongoing effort to develop a standard vocabulary and ontology for describing data sets (see Table 1), which would provide the consistency needed to make repositories more easily searchable.

**Table 1:** Two efforts to develop a standard ontology for describing cognitive neuroscience data

Project name	Goal	Website
Neuroscience Information Framework	A dynamic inventory of web-based neuroscience resources. One goal is to develop and maintain a comprehensive vocabulary for annotating and searching neuroscience resources	http://www.neuinfo.org

Cognitive Paradigm Ontology Project	This project specifically targets the competitive terminologies within cognitive neuroscience research.	http://www.cogpo.org

## Publication worries

Worries that a reanalysis might 'scoop' an important result could be addressed in two ways. First, the researchers who collected the data could, as a standard practice, be offered the opportunity to collaborate on one or more projects that were made possible by sharing the data. This policy would allow the original investigators to be authors on publications resulting from subsequent analyses of their data. In the 'publish or perish' climate that characterizes academic research, this practice could go a long way towards assuaging the fear that sharing fMRI data would translate into fewer publications. Second, researchers could opt to share their data only after they had published their primary findings. These policies would maximize the number of publicly available fMRI data sets while minimizing worries about being scooped.

Worries that a reanalysis might contradict published findings may also influence a researcher's decision to share (or not share) fMRI data. In some cases, such a contradiction might stem from a different method of analysis, in which case it could be highly informative about the phenomenon under investigation. For example, conclusions about the role of visual area V1 in working memory differ depending on whether activity or pattern classification is the dependent measure [15]. In other cases, such a contradiction might result from a genuine error in the original analysis. Nonetheless, reporting such errors would help to ensure that they do not influence the design and analysis of future studies and would likely help the original researchers to avoid similar mistakes in future studies. Thus, reporting discrepancies that are revealed by reanalyzing published data would benefit the cognitive neuroscience community.

## Tools for data sharing

Despite the potential pitfalls associated with sharing fMRI data, some cognitive neuroscientists have already made their data available to the public in various online repositories. Although this practice is quite limited, it is beginning to allow the field to realize some of the potential benefits of more extensive data sharing. We now review these online repositories and provide examples of how they are advancing the field of cognitive neuroscience.

## Repositories for sharing raw fMRI data

These repositories typically include the raw functional and structural images from a study along with meta-data that describes essential aspects of the study. Such meta-data typically include information about how the MRI images were collected, a description of the tasks that were performed by participants, the onset times for different conditions in those tasks, any behavioral data that were recorded, and demographic information about each participant. Armed with this information, researchers can reproduce the published findings or test new hypotheses that were not addressed by the original researchers.

There are several online repositories for raw fMRI data, a few of which are described in Table 2. This admittedly incomplete list illustrates that data sharing is an area of growing interest in cognitive neuroscience. However, it also shows that data sharing has not yet become standard practice in the field, particularly in the realm of task-based studies.

**Table 2:** Some online repositories for raw MRI data

Project name	Description	Website
OASIS project	Anatomical images across the lifespan	http://www.oasis-brains.org

Functional MRI Data Center	Data from task-based fMRI studies (this respository no longer accepts new data sets)	http://www.fmridc.org

Open fMRI Project	Data from task-based fMRI studies (this repository currently has only a few data sets)	http://openfmri.org

1000 Functional Connectomes Project	Data from over 1,400 resting-state functional connectivity data studies are available	http://www.nitrc.org/projects/fcon_1000

We now provide three brief examples to illustrate how sharing raw fMRI data can advance cognitive neuroscience research. First, on a small scale, DHW and colleagues reanalyzed fMRI data from a previous study of the multisource interference task [16] and found that increased reaction time could account for heightened medial prefrontal cortex activity associated with response conflict [17]. This result suggests that any of several processes whose recruitment increases with reaction time (for example, attention, arousal, or effort) might explain such activity and thus raises doubt about the popular claim that such activity is specific to processes that detect response conflict [18]. Second, on a larger scale, a meta-analysis of the hundreds of datasets made available by the 1,000 Functional Connectomes Project revealed that resting-state functional connectivity varies with age, sex, and imaging center [19]. Given that such connectivity often distinguishes patients from healthy controls [reviewed in [20]], further developing our understanding of the variables that influence it may be crucial for interpreting the results of clinical studies. More broadly, such 'mega-analyses' involving hundreds of data sets may provide increased statistical power for revealing the neural substrates of numerous important individual differences. Third, an analysis of 972 data sets from this same repository was recently used to develop a new type of functional connectivity analysis [21]. While by no means exhaustive, these examples illustrate the vast potential of sharing and synthesizing fMRI data to advance the field of cognitive neuroscience.

## Online repositories for sharing processed fMRI data

Repositories of processed fMRI data typically include the peak coordinates (in standardized space) from one or more statistical maps [http://brainmap.org, [22,23]] or sometimes the whole-brain statistical maps themselves [http://sumsdb.wustl.edu/sums, [24,25]]. Each set of coordinates or statistical map is tagged with information about which data analyses were conducted to produce it. Moreover, the data are labeled by the original researchers with the keywords (and ontologies) that are standard to a given databank. This allows other investigators to readily find and retrieve the data from the online repository [23].

Working with processed data allows researchers to quickly combine the results from several datasets [26-29]. Such amalgamations can then be used in meta-analyses to assess the reproducibility of an effect or to determine whether the results of a given study generalize to other paradigms [e.g., [30,31]]. Meta-analyses in which researchers provide peak coordinates from prior experiments are one way to share processed fMRI data [e.g., [32-34]]. However, using searchable databases in combination with statistical methods for aggregating data across studies allows a larger number of datasets to be included in a meta-analysis [14]. Perhaps for this reason, the number of meta-analyses involving searchable databases has grown steadily during the last few years [3,27,29].

Findings from meta-analyses of processed fMRI data can be quite informative. For example, one recent study showed that regions of a so-called 'default-mode network' whose activity is correlated at rest also display correlated activity during task performance [31]. Further, the results suggested regional specialization of function in various regions of the default-mode network. This study provides a simple example of how combining processed fMRI data from a wide range of tasks and subjects, which can be gleaned from an online repository (in this case, the Brainmap database http://www.brainmap.org), can advance the field of cognitive neuroscience.

## Raw data versus processed data

Raw and processed data offer complementary strengths and weaknesses. Raw data permit a researcher to test a wider range of novel hypotheses, but take time to analyze. Processed data allow a researcher to quickly compare the results of numerous studies, but offer less flexibility with regard to testing new hypotheses. Thus, a researcher's choice about whether to use raw or processed data should probably be made while considering the goals of the study.

If a researcher chooses to reanalyze processed fMRI data, then he or she should be aware of several factors that might limit the conclusions that can be drawn. As an illustration, consider a hypothetical meta-analysis of medial prefrontal cortex activity across 50 studies. Differences in the location of such activity could be due to the factors of interest that distinguish the studies (for example, the tasks performed, the subjects involved, and so on). On the other hand, they could be due to differences in the analyses and/or statistical thresholds that different researchers employed. In short, although processed fMRI data are preferable to raw fMRI data in some situations (for example, a meta-analysis), they sometimes limit the conclusions that researchers can draw.

## Summary

Analogous to data sharing in other fields, sharing fMRI data offers many potential advantages to the field of cognitive neuroscience. These include quickly establishing the reproducibility of a new finding, allowing researchers with complementary expertise to provide multiple characterizations of the same data, and enhancing the training of new students. Although there are some potential drawbacks, we feel that many of these can be ameliorated through continued discussion and development of consensus in the cognitive neuroscience community. Thus, we conclude that the field should strive to overcome these potential pitfalls so that it can grow to fully realize the benefits of sharing fMRI data.

## Text box: The 'discovery' of pattern classification techniques

In recent years, pattern classification techniques for analyzing fMRI data have greatly influenced cognitive neuroscience research. These techniques enable researchers to determine whether the spatial distribution, or pattern, of activity varies for different conditions or stimuli, consistent with the idea that information is distributed in the brain [35]. They also provide greater sensitivity for identifying differences in activity between two conditions than does contrasting activity for those conditions in isolated regions [3].

**Figure 1:**
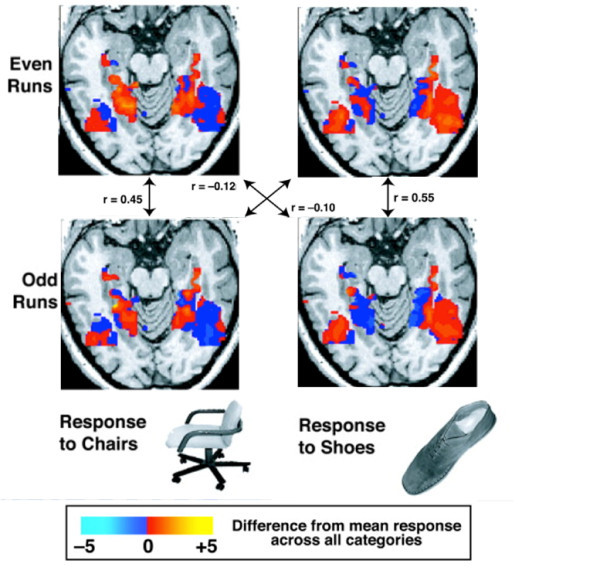
**Patterns of activity in the visual cortex**.

Pattern classification techniques were 'discovered' when cognitive neuroscientists realized that they had been applied in other fields to solve complex problems such as face recognition [5], handwriting recognition [36], and the analysis of DNA microarray data [37]. Soon afterward, cognitive neuroscientists began using these techniques on fMRI data with great success [35,38-40]. For example, an early study showed that different 'patterns' of activity in the visual cortex were evoked by different types of objects (for example, chairs and shoes), and that this effect was not simply due to regional differences in activity for those objects [35]. As shown in Figure 1, the pattern of visual cortex activity evoked in different parts of the study (even runs versus odd runs) was more similar (that is, highly correlated, as indicated by higher r-values) when the same type of object was presented (see vertical lines) than when a different type of object was presented (see diagonal lines).

As mentioned above, pattern classification techniques were applied to fMRI data only after cognitive neuroscientists realized they were useful in other fields. By allowing researchers with different areas of expertise to work directly with raw fMRI data, greater data sharing would distribute the process of developing and testing new analytic methods across a much wider variety of individuals and laboratories. Such a distribution could more quickly reveal which methods from other fields are likely to be useful, thereby increasing the rate at which new discoveries are made in cognitive neuroscience.

## Competing interests

The authors declare that they have no competing interests.

## Authors' contributions

DHW and KMV contributed equally to the work of researching and writing this article.

## Pre-publication history

The pre-publication history for this paper can be accessed here:

http://www.biomedcentral.com/1741-7015/9/34/prepub
